# Mixture Optimization of Recycled Aggregate Concrete Using Hybrid Machine Learning Model

**DOI:** 10.3390/ma13194331

**Published:** 2020-09-29

**Authors:** Itzel Nunez, Afshin Marani, Moncef L. Nehdi

**Affiliations:** Department of Civil and Environmental Engineering, Western University, London, ON N6G 1G8, Canada; inezvarg@uwo.ca (I.N.); amarani@uwo.ca (A.M.)

**Keywords:** recycled aggregate concrete, machine learning, model, Gaussian process, deep learning, gradient boosting, regression trees, gated recurrent unit

## Abstract

Recycled aggregate concrete (RAC) contributes to mitigating the depletion of natural aggregates, alleviating the carbon footprint of concrete construction, and averting the landfilling of colossal amounts of construction and demolition waste. However, complexities in the mixture optimization of RAC due to the variability of recycled aggregates and lack of accuracy in estimating its compressive strength require novel and sophisticated techniques. This paper aims at developing state-of-the-art machine learning models to predict the RAC compressive strength and optimize its mixture design. Results show that the developed models including Gaussian processes, deep learning, and gradient boosting regression achieved robust predictive performance, with the gradient boosting regression trees yielding highest prediction accuracy. Furthermore, a particle swarm optimization coupled with gradient boosting regression trees model was developed to optimize the mixture design of RAC for various compressive strength classes. The hybrid model achieved cost-saving RAC mixture designs with lower environmental footprint for different target compressive strength classes. The model could be further harvested to achieve sustainable concrete with optimal recycled aggregate content, least cost, and least environmental footprint.

## 1. Introduction

Portland cement concrete is the most widely used construction material and the most consumed industrial product in the world. However, the more frequent local shortages of natural aggregates (NA) and the enormous environmental footprint caused by its extraction are global concerns regarding the production of Portland cement concrete. The global annual consumption of NA is estimated at 8 to 12 billion tons [1,2,3].

Moreover, there has been a growing worldwide shortage of available landfilling sites to dispose of construction and demolition waste (CDW). In Canada, it is estimated that 9 million tons of CDW are produced every year. Despite the vast country, its biggest cities are encountering stern CDW disposal challenges [4]. Likewise, several reports are forecasting that landfills in Hong Kong will be overfilled in about eight years [2]. The use of recycled aggregate (RA) offers a potential solution to overcome the drawbacks of concrete production. Among the most promising advantages of RA are reductions in CO_2_ emissions and beneficiation of CDW in applications with added value. About 75% of construction waste, including concrete and masonry, can be reused in concrete production [5].

However, the inclusion of RA in concrete has been reported to reduce the compressive strength [6]. Several researchers have explored the influential factors on recycled aggregate concrete (RAC) compressive strength [7,8,9,10]. The moisture content of RA, replacement level of NA, and the water-to-cement (w/c) ratio were found as the parameters with the highest effect on RAC compressive strength [10]. The higher absorption capacity of RA compared to that of NA, along with the relatively weaker interfacial bond between RA and the cementitious matrix are major explanations for such a behavior [9,11,12].

Although there have been multiple studies on the mechanical properties of RAC, more research needs to be devoted to investigating the effects of various parameters on RAC compressive strength, including the variability of RA, the effect of old cement mortar attached to the RA surface, and the crushing process of RA [7,13]. Considering the fundamental knowledge gaps in the mechanical, durability, and structural performance of RAC, its application has generally been limited to road bases and non-structural concrete [6,8]. Hence, it is of paramount importance to conduct comprehensive experimental studies on RAC, develop pertinent standards and guidelines, and deploy advanced practical frameworks to promote its wider utilization.

The lack of understanding of RAC has resulted in developing new modeling techniques, such as machine learning (ML) algorithms, to predict its mechanical properties. A major advantage of ML methods is that they can capture the underlying mechanisms, despite the lack of information regarding specific parameters. Data driven ML techniques have proven to be successful in the prediction of RAC mechanical properties including the modulus of elasticity and compressive strength [11,14,15,16]. Nevertheless, most research studies employed small datasets, which compromises the training of such models and the ability to generalize their predictions to new data unseen to the model. Thus, creating reliable and more comprehensive data has been regarded as a key research need, and several studies aimed at deploying larger datasets for better generalization and robustness of the RAC-ML models.

ML techniques have also been employed for mixture design and optimization. Concrete mixture design is the process of selecting the appropriate quantitative proportions of concrete ingredients [17]. From a computational point of view, mixture optimization is the process of minimizing a prior defined objective function [18]. A common practice in concrete mixture optimization procedures is to consider the concrete production cost function as the objective function [19,20,21]. Moreover, the current stringent mechanical requirements for concrete should be met in the optimization process. Hence, in this study, the particle swarm optimization (PSO) algorithm was used to execute the mixture optimization. Subsequently, to assure that the compressive strength was met, the best performing ML model was used to predict the compressive strength of the RAC.

Accordingly, the present study aims at creating a large and comprehensive experimental dataset from the available studies in the open literature to develop powerful state-of-the-art ML models to predict the compressive strength of RAC. For this purpose, a dataset consisting of 1134 experimental examples of RAC mixture designs with 10 attributes was developed. Moreover, three different novel ML models were developed, and their performance was compared. Gaussian processes (GP), deep learning (DL) and gradient boosting regression trees (GBRT) techniques were employed for the first time to model the compressive strength of RAC. Eventually, an optimization of the RAC mixture design was performed by coupling PSO with the best proposed ML model to develop a hybrid powerful model for optimizing RAC mixture composition for different target ranges of compressive strength at 28 days. The superior accuracy of the proposed models should assist various stakeholders in optimal use of recycled concrete in diverse construction applications.

### 1.1. Related Work

Other studies have employed ML to predict the compressive strength of RAC. For instance, Khademi et al. [15] used three different approaches to model the compressive strength of RAC: artificial neural network (ANN), adaptive neuro-fuzzy inference systems (ANFIS), and multiple linear regression. They used 14 different input parameters, including the dosage of concrete ingredients and non-dimensional parameters such as the water-to-cement ratio and aggregate-to-cement ratio. It was concluded that multiple linear regression might be inaccurate to determine the compressive strength of RAC due to the highly non-linear relationships between the concrete ingredients and its strength. However, both ANN and ANFIS models proved to be powerful in modeling the compressive strength of RAC, with a coefficient of determination of 0.9185 and 0.9075 for ANN and ANFIS, respectively. Furthermore, Khademi et al. [15] performed a sensitivity analysis in which they concluded that the inclusion of more input features resulted in higher model predictive accuracy. Likewise, Naderpour et al. [1] developed an ANN model to predict the compressive strength of RAC with a coefficient of determination of 0.829 for the testing dataset. They also performed a sensitivity analysis via the weights of the input features. Accordingly, it was found that the water absorption of aggregates and the water-to-total materials mass ratio had the highest importance. In another study, Deng et al. [16] built a convolutional neural network to predict the compressive strength of RAC. Experimental work was carried out along with the development of the deep learning model. The authors compared the convolutional neural network with a support vector machine and a back propagation neural network concluding that the convolutional neural network has superior capability to predict the compressive strength of RAC. They used the relative error percentage to measure the performance of the models, and thus the error for the convolutional neural network, back propagation neural network and support vector machine was 3.65%, 6.63%, and 4.35%, respectively. Deshpande et al. [11] compared three different techniques: ANN, model tree, and non-linear regression. They studied the influence of adding non-dimensional parameters as input features. To accomplish such analysis, they created 10 different models for each algorithm and added a different non-dimensional input feature to the parameters corresponding to the ingredients content. The accuracy of the ANN model was at least 2% higher than that of the other techniques, even when the non-dimensional parameters were considered. Using a larger dataset, Gholampour et al. [22] predicted the compressive strength and other mechanical properties of RAC employing three types of algorithms including multivariate adaptive regression splines, M5 model tree, and least squares support vector regression. They created two different models for each algorithm corresponding to the cube and cylinder compressive strengths, respectively. For these models, results on 332 cube-specimens and 318 cylinder-specimens were collected from the open literature. It was found that least squares support vector regression achieved higher performance than the remaining models, with at least 12.6% better mean absolute percentage error. Duan et al. [2] proposed using the characteristics of the recycled aggregate as input parameters, including the saturated surface dry mass, water absorption, and volume fraction of coarse aggregate. They concluded that the inclusion of these features had a positive effect on model accuracy. Moreover, Topcu and Saridemir [6] found that ANN had better predictive accuracy than of the RAC compressive and splitting tensile strengths than fuzzy logic. The ANN model demonstrated to be a powerful tool to determine the mechanical properties of RAC, achieving a coefficient of determination of 0.9984 and 0.9979 for the prediction of compressive strength and splitting tensile strength, respectively. Dantas et al. [23] gathered the largest dataset and used ANN to develop an equation to describe the compressive strength of RAC. Their model included 17 input features, from which, the ratio of recycled concrete, absorption rate of fine recycled aggregate, content of dry aggregate, and finesses modulus of aggregates were the parameters with highest effect on the compressive strength of RAC. The reported accuracy for the training and testing sets were 0.928, and 0.971, respectively.

In summary, Khademi et al. [15], Naderpour et al. [1], Deng et al. [16], Deshpande et al. [11], Gholampour et al. [22], Duan et al. [2], Topcu and Saridemir [6], and Dantas et al. [23] used 257, 139, 74, 257, 650, 168, 210, and 1178 data points, respectively, to predict the compressive strength of RAC. In addition to the quality and size of existing datasets, the advent of new and more powerful ML algorithms has stimulated researchers to explore the ability of state-of-the-art methods to enhance the accuracy and robustness of predictive models. Among various ML techniques to predict the compressive strength of RAC, artificial neural networks (ANNs) and fuzzy logic have been the most widely applied methods (Table 1).

### 1.2. Research Significance

As elaborated on above, there have been various studies on the application of traditional ML techniques to predict the compressive strength of RAC. The present study aims at creating a large and more comprehensive dataset and deploy it with state-of-the-art ML techniques that have not yet been explored for RAC in the open literature. The models presented herein are executed using the Python programming language. Therefore, to utilize these models, the user can simply apply the development steps along with hyperparameters reported in this study. Furthermore, the compressive strength predictive tools developed in this study are further complemented with optimization of the mixture proportions using a coupled PSO-GBRT model. The proposed mixture proportions can be used as a reference guideline for designing eco-friendlier and more economical RAC mixtures in practice.

## 2. Machine Learning Basis

ML refers to a computer’s capacity of analyzing data and learning complex patterns within the data without being rigorously programmed [24]. Depending on the nature of the data, ML algorithms are categorized into supervised learning, unsupervised learning, and reinforcement learning [25]. Supervised learning aims at capturing underlying patterns in the data with known outputs. Depending on the type of the output, it can be further categorized as classification for discrete outputs, and regression for continuous outputs. Unsupervised learning, on the other hand, is associated with the data with unknown outputs and thus, clusters the data by finding relationships within the observations [26]. The third type of machine learning, reinforcement learning, bridges the gap between supervised and unsupervised learning since it clusters similar data given the correct answers [27]. Three powerful ML models were developed herein to forecast the compressive strength of RAC: GP, recurrent neural networks (RNNs), and GBRT. The three algorithms have different approaches for data analysis. Whilst GBRT is an ensemble of decision trees, GP uses the Gaussian distribution, and finally, RNNs are an advanced type of neural networks. The diverse nature of these algorithms is considered to explore the robustness of ML algorithms. The fundamentals of GP, RNNs, and GBRT are discussed below.

### 2.1. Gaussian Processes

GP are stochastic processes that generalize the Gaussian probability distribution [28]. In contrast to single- or multi-variable probability distribution in which a scalar or a vector is mapped, a process describes the properties of functions [29]. Therefore, a GP is defined as a probability distribution of functions, P(f), where P(f) has a Gaussian distribution [30]. GPs are parametrized with mean and covariance by analogy with Gaussian distribution, whereas mean and covariance for GP are functions [31]. The purpose of training a supervised learning algorithm using the available training dataset is to develop a model capable of predicting the output for unseen input data. There are two common approaches to determine the appropriate function that fits a set of data with promising accuracy [29]. In the first approach, the model is generated by considering only certain types of functions, e.g., exponential functions [29]. However, the prediction accuracy of such models strongly depends on the performance of the given functions. Conversely, the second approach considers pre-assigned probabilities of several types of functions such that higher probability is assigned to those that are more likely to predict with higher accuracy [32]. The complexity of the first approach is limited to the selected functions. By contrast, the second approach is computationally more costly since there is an infinite number of possible functions to consider [29]. GPs are based on the second approach. The probabilistic formulation of GPs gives rise to a phenomenon called computational tractability in which the properties of functions are inferred even when some of the functions are ignored [33].

### 2.2. Recurrent Neural Networks

DL models are multiple-level computational algorithms able to learn complex underlying structures within a database [34]. DL models have proven successful in diverse applications such as image recognition, language understanding, and deoxyribonucleic acid (DNA) biological processes prediction [34]. However, applications of recent DL algorithms in civil engineering, including convolutional neural networks (CNNs) and RNNs have been more common in structural health monitoring and crack detection owing to the large data sets available in these fields [35,36]. CNNs and RNNs are among the most popular DL algorithms. In the present study, a novel type of RNN is deployed to predict the compressive strength of RAC.

**Figure 1:**
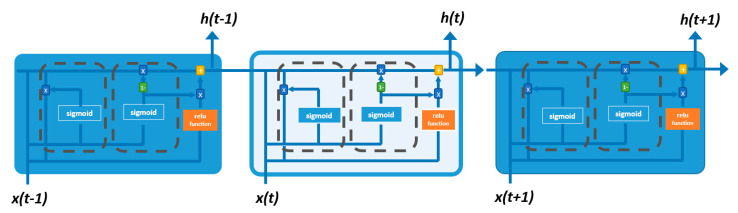
RNN structure using one GRU hidden layer.

**Figure 2:**
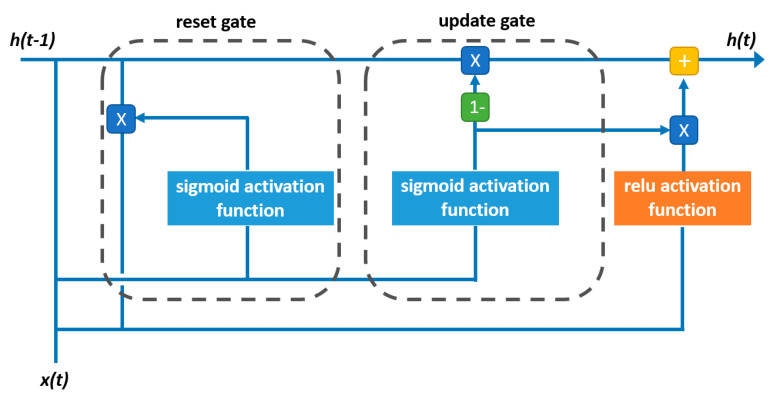
GRU hidden state computation, corresponding to the first layer of the developed deep learning model.

RNN is a class of neural networks with an internal loop that allows the algorithm to keep memories from past information, commonly referred to as hidden state [37,38]. In RNNs, the output of a certain step, t, is used as input for the next step, t+1, emphasizing that every single step is based on the previous one, a process referred to as long-term dependencies; see Figure 1 [37]. Simple RNNs have a limitation regarding the contribution of earlier steps to the later ones, known as vanishing gradients [37]. Two main variants of layers have been proposed for RNN to overcome vanishing gradients: long-short-term memory (LSTM) and gated recurrent unit (GRU) [38]. The main difference of these RNN algorithms is the inclusion of gates for computing data. For example, LSTM layers incorporate a third gate, named the forget gate, in addition to the input and output gates in the simple RNN [37]. The forget gate maintains information and includes it in a non-consecutive step [38]. Conversely, GRU layers have only two types of gates: reset gate and update gate. In the reset gate, the previous information is combined with the most recent information, whereas in the update gate, it is decided how much information is to be passed to the following step. Figure 2 displays the structure of the first GRU layer used in this study [37]. Like LSTMs, GRUs are not affected by vanishing gradients. Nonetheless, GRU is considered a more efficient algorithm due to its simpler structure and formulation [27]. The formulation of GRU is summarized in the following:

(1) $$r_{t}=\sigma \left(W_{r}\;x_{t}+U_{r}\;h_{t-1}\right)$$

(2) $$z_{t}=\sigma \left(W_{z}\;x_{t}+U_{z}\;h_{t-1}\right)$$

(3) $$\bar{h_{t}}=ReLu\left(W_{h}\;x_{t}+U_{h}\;\left(r_{t}\times h_{t-1}\right)\right)$$

(4) $$h_{t}=\left(1-z_{t}\right)\times h_{t-1}+z_{t}\times \bar{h_{t}}$$

where and $r_{t}$ are the reset and update gate, respectively, $\bar{h_{t}}$ is the candidate output, and $h_{t}$ is the corresponding output of the cell for the time step t. Accordingly, $W_{r}$, $W_{z}$, $W_{h}$, $U_{r}$, $U_{z}$, and $U_{h}$ are the weight matrices that operate the input vector $x_{t}$ and the previous state $h_{t-1}$, and ReLU is the rectified linear unit activation function [39,40].

### 2.3. Gradient Boosting Decision Trees

GBRT algorithm integrates multiple weak learners using a boosting approach in which additional trees are appended in sequence without model parameters being changed. The objective of the gradient boosting is to find the function ℱ(X) that minimizes the loss function ℒ(ℱ(X),Y) (e.g., mean squared error or mean absolute error) using a given dataset, $\left\{\left(x_{1},y_{1}\right),\;\left(x_{2},y_{2}\right),\;\ldots ,\left(x_{N},y_{N}\right)\right\}$ [41,42]. The predictions of the GBRT model, $y_{t}$ for a given input data can be expressed as:

(5) $$y_{t}={ℱ}_{m}\left(X_{t}\right)=\overset{M}{\underset{m=1}{\sum }}{𝒽}_{m}\left(x_{t}\right)$$

where the ${𝒽}_{m}$ are referred to as weak learners. The constant *M* represents the number of weak learners which is known as the n_estimators hyperparameter. The loss function represents to what extent the predicted value is close to the output in the dataset using a specific metric. GBRT approaches the best function using the weighting of weak learner models, $𝒽\left(x_{t}\right)$, which is the basic decision tree fit by the input variables and the negative gradient of the last model’s loss function. GBRT develops the model in a greedy manner considering a constant initial function $F_{0}\left(X\right)$ as follows [41,42,43,44]:

(6) $$F_{0}\left(X\right)=argmin\overset{N}{\underset{t=1}{\sum }}ℒ\left(y_{t},\gamma \right)$$

(7) $${ℱ}_{m}\left(X\right)={ℱ}_{m-1}\left(X\right)+\gamma_{m}{𝒽}_{m}\left(x\right)$$

where ${𝒽}_{m}\left(x\right)$ is the mth regression tree and $\gamma_{m}$ is its weighting coefficient, also called learning rate. In a GBRT model, the number of trees, the learning rate, and the max depth of the tree are amongst the most essential hyperparameters that noticeably affect the predictive performance of the model. Larger number of trees increases the prediction accuracy of the model; however, excessive trees could result in an over-fitted model with lack of generalization for new unseen data. On the other hand, the learning rate controls the contribution of each tree to the predictions, while the max depth indicates the complexity of each tree. Immoderate values of such hyperparameters could bring about either over-fitted or erroneous models [41,42,43,44]. Other parameters of the GBRT model, such as subsample, maximum number of features, etc., also have noticeable effects on the model output and should be considered. Hence, tuning the GBRT hyperparameters is essential to propound robust and reliable performance.

## 3. Dataset Creation and Model Development

### 3.1. Data Collection and Preprocessing

**Figure 3:**
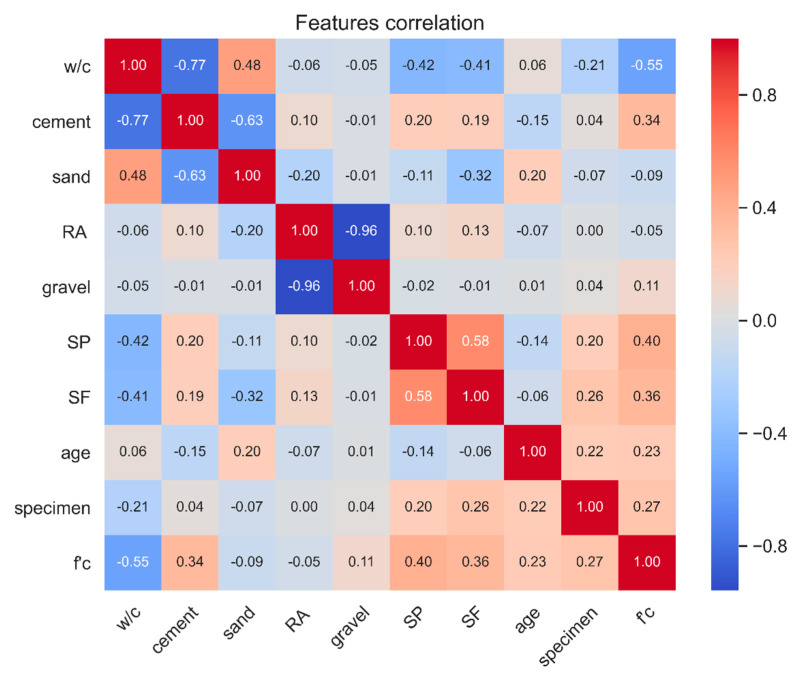
Pearson correlation coefficient for the dataset attributes.

The experimental data used in this study was collected from 55 peer-reviewed publications (Table 2). The collected data consists of 1134 RAC mixture design examples, with nine input features and one output. Statistical characteristics of the dataset are given in Table 3. Figure 3 illustrates the Pearson correlation coefficient between different attributes of the dataset. The Pearson correlation coefficient is an indicator of linear dependencies within two random variables; i.e., a coefficient of correlation close to one within two variables indicates that an increase in one of those variables will result in a proportional increment of the other [45]. Accordingly, the w/c ratio and superplasticizer dosage were the features with the highest correlation to the compressive strength. Conversely, aggregates (sand, natural gravel and RCA) did not have significant linear correlation to the compressive strength. Furthermore, since gravel is an ingredient replaced by recycled coarse aggregate (RCA), there was a high correlation between these two features.

Feature normalization is commonly applied prior to modeling. Although normalization is not required for all ML algorithms, it has been proven to improve the model performance [27]. Linear transformation and statistical standardization are among the most popular normalization techniques [98]. In the linear transformation, values are ranged within the domain of [0, 1], whereas in the statistical standardization, the mean and the standard deviation values of the data are set equal to 0 and 1, respectively [98]. In this study, statistical standardization was used prior to GP, GBRT and RNNs modeling. The data was then randomly divided into training and testing sets using 70% (793 samples) for training and the remaining (341 samples) for model testing.

**Figure 4:**
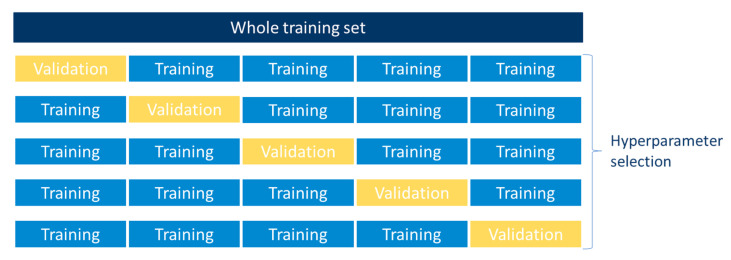
5-fold cross validation for hyperparameter tuning.

A common practice to assess the performance of ML models is to divide the entire dataset into three different subsets: training, validation and testing. Whilst the learning process is accomplished with the training set, the validation set is used to track the performance of the model, while the testing set serves to assess the extrapolation capabilities of the model by performing it over unseen samples that are not known to the model [27]. However, the partition of data into three subsets leads to a reduction of the training samples, which consequently can result in an insufficiently trained model [27]. Thus, cross-validation is a common technique to prevent the over reduction of the training set, especially for small datasets. There are several techniques to perform cross-validation, most of which consist in leaving out random data to validate the model [99]. In this study, *K*-fold cross-validation was used. *K*-fold cross-validation is a resampling method that splits the data into *k* number of subsets and keeps one subset for validation, while the other k−1 subsets are used for training [100]. The 5-fold cross-validation employed for hyperparameter selection in this study is schematically depicted in Figure 4.

### 3.2. Hyperparameter Tuning

Hyperparameter tuning is a crucial step in developing robust ML models. Tuning of ML model mitigates over-fitting and thus, enhances the versatility of the model to unseen data [101]. The selection of optimum hyperparameters is also a determinant factor in increasing the model accuracy [102]. Aiming to avoid manual tuning, there have been different approaches proposed to automize the selection of hyperparameters, such as grid search and random search hyperparameter optimization [103]. These approaches are distinct from each other by the domain of the potential values considered in the search attempt. Whilst grid search explores all possible values in a pre-defined domain for hyperparameters, random search algorithms select the different hyperparameter values in a random manner for a specific number of iterations [103]. A randomized search procedure with 5-fold cross-validation was used herein for exploring possible values of hyperparameters using the Scikit-learn package in Python [104].

### 3.3. Model Development

#### 3.3.1. GP Model

GP is a non-parametric model [29] and thus, the selection of hyperparameters is less challenging, especially compared to DL models. The hyperparameters of GP models are those required for the kernel function. Therefore, the kernel function, also known as the covariance function, is key to creating robust GP models [29]. In this study, a linear combination of several default kernel functions was implemented as defined in Equation (8). This kernel function includes the periodic kernel, matern kernel, and dot-product kernel. It is worth mentioning that all available kernels, such as the periodic kernel, the rational quadratic kernel, white kernel, matern kernel, and dot-product kernel, were tested for tuning the GP model.

(8) $$\begin{array}{cc} k\left(x_{i},x_{j}\right)= & \sigma_{0}^{2}+x_{i}\cdot x_{j}+2^{2}*\exp\left(-\frac{2\;\sin^{2}\left(\pi \frac{d\left(x_{i},x_{j}\right)}{p}\right)}{l_{1}^{2}}\right)\\ & +\frac{1}{\Gamma \left(\nu \right)2^{\nu -1}}{\left(\frac{\sqrt{2\nu }}{l_{2}}\;d\left(x_{i},x_{j}\right)\right)}^{\nu }K_{\nu }\left(\frac{\sqrt{2\nu }}{l_{2}}\;d\left(x_{i},x_{j}\right)\right) \end{array}$$

According to the former equation, parameters associated with the considered kernels were tuned as the hyperparametrs of the GP model, including the length scale 1 ($l_{1}$) and periodicity (p) corresponding to the periodic kernel; ν and length scale 2 ($l_{2}$) corresponding to the matern kernel; and $\sigma_{0}$ of the dot-product kernel. The optimizing of hyperparameters was carried out using 5-fold cross-validation (CV) as described earlier. The tuned values of the hyperparameters are listed in Table 4. Scikit-learn library in Python was employed for tuning and executing the GP model [104].

#### 3.3.2. RNN Model

The developed architecture of the RNN model consists of 3-GRU layers and 1 dense layer with 239, 238, 217, and 1 hidden neuron, respectively. In the first layer, ReLU activation function and sigmoid recurrent activation function were utilized (Figure 2). In the second layer, the activation function and recurrent activation function were the sigmoid and ReLU, respectively. In the third layer, the scaled exponential linear unit (SELU) and softsign were used as activation and recurrent activation functions, respectively. For the dense layer, only the softplus activation function was used. Moreover, the kernel initializer and recurrent initializer were tuned for GRU layers. The kernel initializer was fixed as random uniform for the first and second layers, whereas a constant initializer was used for the third layer. The recurrent initializer was set as constant for the first layer, and zero recurrent initializer for the second and third layers. Mean squared error (MSE) was used as the model loss function, whereas the Adam optimization algorithm was employed as the model optimizer, with a learning rate of 0.0002. Ultimately, the number of epochs and batch size were set to 360 and 11, respectively. According to Whang and Matsukawa [105], the performance of GRU models was improved when batch normalization was applied. Batch normalization mitigates the so-called internal covariate shift [105]. Internal covariate shift is a frequent problem in the training step of deep neural networks in which the distribution of inputs at each layer is changed and thus, finer tuning for models along with smaller learning rates are required [106]. Hence, batch normalization was implemented in the developed RNN model because it improves the performance of GRU networks [105]. Momentum and epsilon are the parameters associated with batch normalization. The optimum momentum and epsilon were found to be 0.95 and 0.0001, respectively. Table 5 summarizes the tuned hyperparameters of the RNN model. The hyperparameter selection for the DL models was performed using a randomized search approach along with 5-fold CV. Keras API and Scikit-learn packages in Python were utilized for building and tuning the RNN model [104,107].

#### 3.3.3. GBRT Model

GBRT has multiple hyperparameters that need tuning prior to model training. In the current study, a randomized search procedure alongside 5-fold CV was used to obtain optimum hyperparameters of the GBRT model. Generally, n_estimators and learning_rate, which indicate the number of the weak learners in the model and the weighting of each estimator, respectively, are the most influential hyperparameters of the GBRT model that are essential to be tuned. Additionally, max_depth, max_features, and subsample can greatly affect the prediction performance of the GBRT model [108]. Table 6 presents the tuned values of the seven hyperparameters considered. The mean absolute error (MAE) was monitored as the statistical error to achieve optimum hyperparameters yielding highest accuracy while mitigating over-fitting. The Scikit-learn package was implemented to perform GBRT modeling and tuning [104].

### 3.4. RAC Mixture Optimization

This section presents the framework adopted for optimizing the mixture design of RAC using the ML model with best predictive performance. The objective of the optimization is to propose the most economic mixture proportions of RAC considering different classes of compressive strength. The PSO algorithm, which is a metaheuristic method that mimics the social interactions of birds or insects (particles) in the search of an optimal solution, was adopted [109]. The particles modify their position in every iteration based on the individual velocity vector of each particle, which in turn is dependent on both the best-found particle and swarm positions [110]. The PSO minimizes an objective function while limiting the domain of the solution. According to the optimization procedure proposed by Yeh [19], the function that is to be optimized herein is the cost to produce a batch of RAC as defined in Equation (9). The considered unit costs, which are averages of values retrieved from multiple material suppliers across Canada, are presented in Table 7. These values can easily be replaced by costs corresponding to other locations. The unit cost of RCA was considered equal to that of NA as recommended in ref. [111].

(9) $$P=C_{1}\;I_{1}+C_{2}\;I_{2}+\cdots +C_{i}\;I_{i}$$

were $C_{i}$ represents the unit cost of the ith ingredient of the mixture and $I_{i}$ is the ith ingredient dosage in $kg/m^{3}$. To limit the domain of the solution, two bounder vectors were defined: upper limit and lower limit. The bounder vectors (Table 8) were strategically defined based on a real experiment from the dataset with certain compressive strength to draw meaningful comparison and thus, better validate the performance of the algorithm. In other words, for sand, cement, and water, the upper and lower bounder limits were defined in average 20% up and down the values given for the base mixture. To promote the use of recycled aggregate, the assigned values to the lower and upper bounder vectors were kept high, and the corresponding values for gravel were maintained low. Also, due to the high cost of superplasticizer, the assigned values for the bounder vectors were kept as low as possible. The 28-day compressive strength of a standard 15 × 30 cm cylinder specimen was considered for the sake of comparison. The results of the optimized mixture proportions are given in Table 9. The optimized mixture was subsequently tested using the GBRT (being the best predictive model in this study) and compared to the real concrete sample extracted from the dataset to ensure the required compressive strength criteria, as shown in Table 10.

## 4. Results, Discussion, and Recommendations

This section presents the results of ML modeling of RAC. The three different models outlined earlier were implemented and their prediction performance is discussed herein. Purposefully, the root mean squared error (RMSE), mean absolute error (MAE), and coefficient of determination $R^{2}$ were used for assessing the performance of each model. Moreover, the best acquired ML model was employed to perform RAC mixture design optimization for different ranges of 28-day compressive strength. The optimization results along with mixture proportion recommendations are discussed below.

### 4.1. Prediction Performance of ML Models

**Figure 5:**
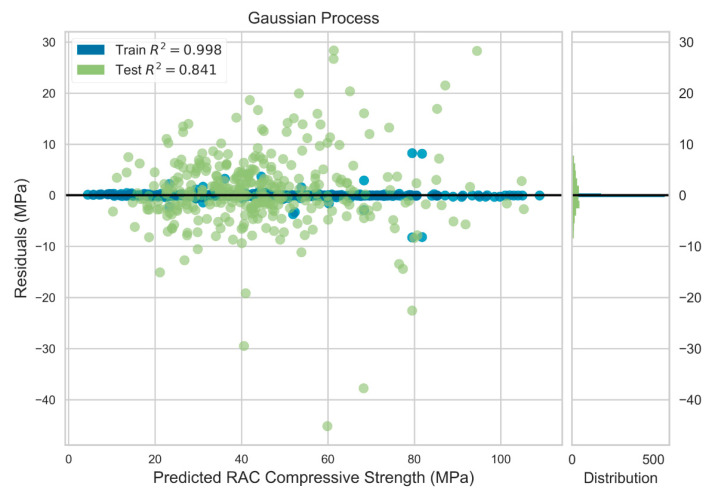
Residuals plot for Gaussian process model.

**Figure 6:**
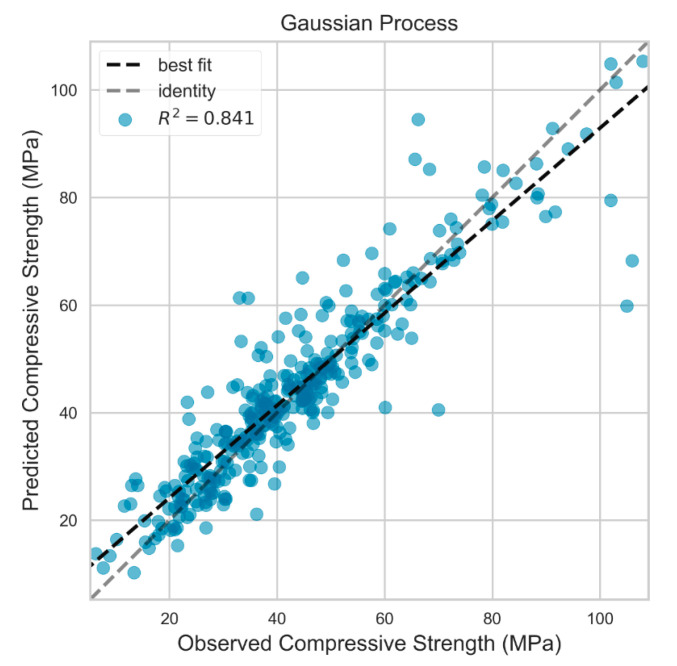
Actual vs. predicted values for testing set in Gaussian process model.

GP, GRU, and GBRT models were trained using 793 data examples and tested with the remaining 341 data. The final tuned models were executed over five different seed numbers of data split to assess the robustness of the models trained with randomized split of the data for training and the testing sets. The predictive performance of the GP model for the five random seed numbers is summarized in Table 11. The model predicted the output with average RMSE, MAE and $R^{2}$ of 7.087 MPa, 4.911 MPa, and 0.844, respectively for the test dataset. However, the model performace was greatly superior for the training dataset with average RMSE, MAE and $R^{2}$ of 0.735, 0.138, and 0.998, respectively. This trend can be further observed in the residual plot of the GP model shown in Figure 5. The residualts for the training data were less than 10 MPa, while they were as high as 40 MPa for some data points in the testing set. The actual versus predicted output of the GP model is illustrated in Figure 6.

**Figure 7:**
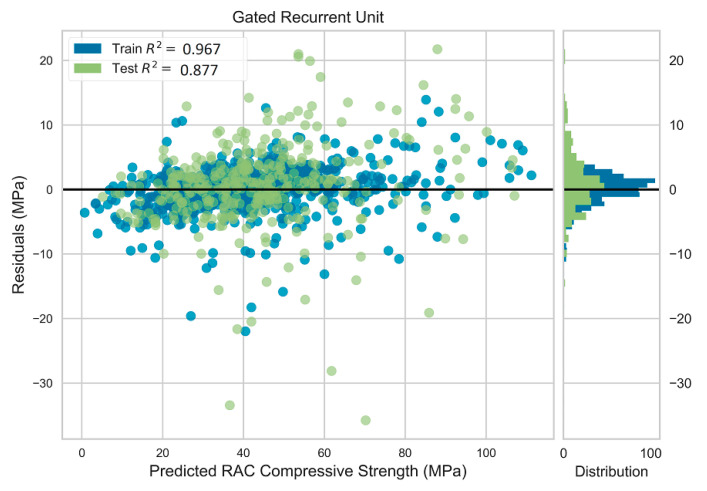
Residuals plot for deep learning model.

**Figure 8:**
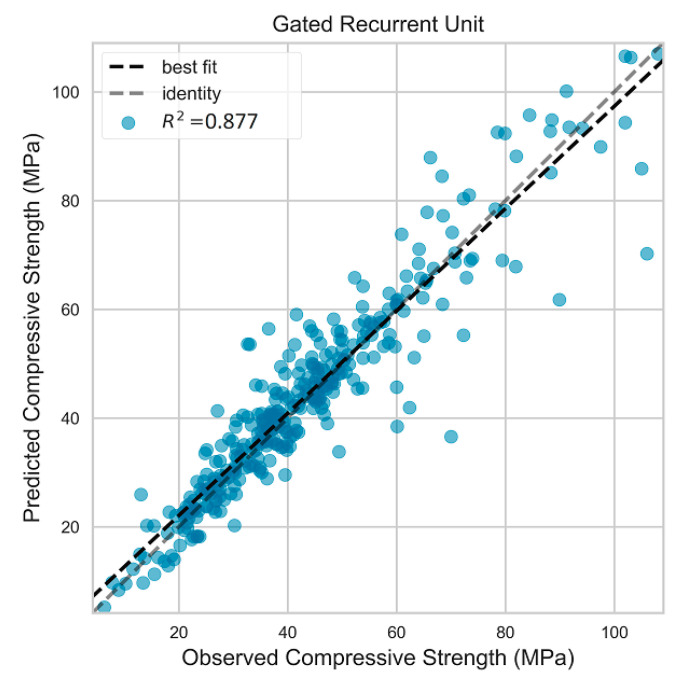
Actual vs. predicted values for testing set in deep learning model.

The GRU model attained better performance compared to that of the GP model (see Table 12). The difference between the GRU statistical errors of train and test data were less than that of the GP model. The RMSE, MAE, and $R^{2}$ values for the test dataset were 6.502 MPa, 4.364 MPa, and 0.868, respectively, while the corresponding values were 3.183 MPa, 2.285 MPa, and 0.968, respectively, for the train dataset. This demonstrates more robust predictive performance along with higher accuracy compared to the GP model. The residuals of the predictions varied in a narrower range compared to that in the GP model, as depicted in Figure 7. The residuals for both testing and training datasets had similar normal distribution, indicating more robust predictive performance. Figure 8 shows the actual versus predicted compressive strength of the test data for the GRU model.

**Figure 9:**
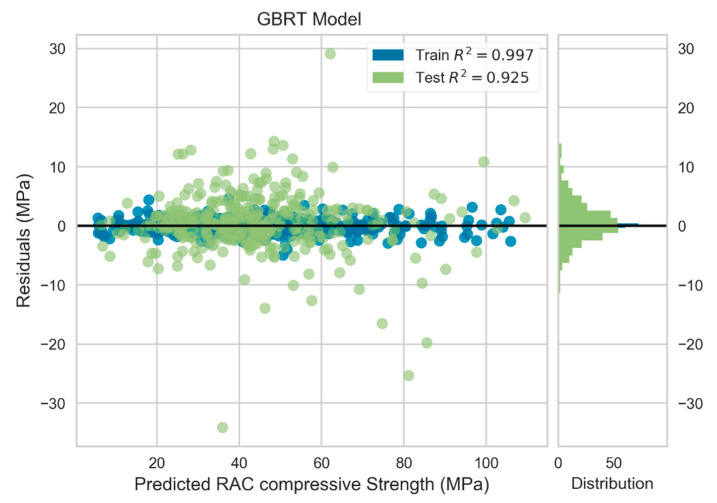
Residuals plot for GBRT model.

**Figure 10:**
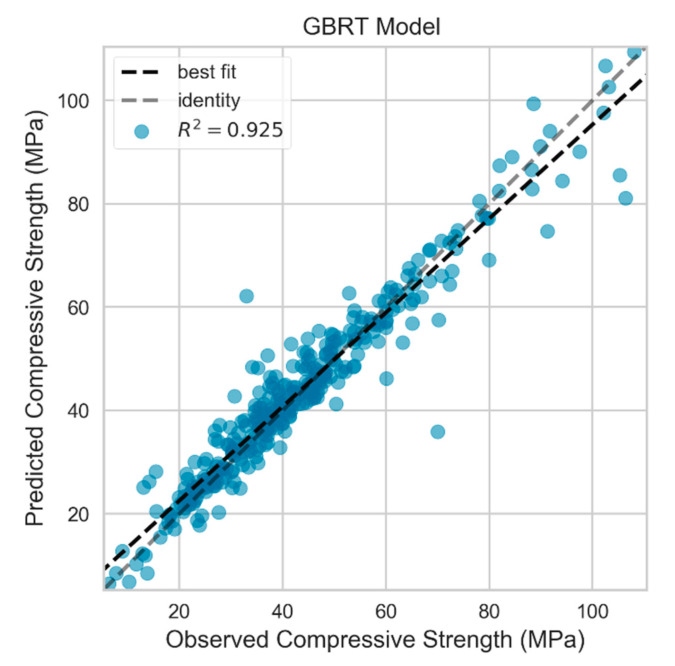
Actual vs. predicted values for testing set in GBRT model.

The GBRT model scored superior predictive execution, as indicated in Table 13, with lowest RMSE and MAE values for the test dataset, along with the highest coefficient of determination compared to that of the GP and GRU models. RMSE and MAE were 5.074 and 3.396 MPa, respectively for the GBRT model. Figure 9 depicts the residuals of the predicted compressive strength for the training and testing datasets of the GBRT model. It can be observed that the model captured the trend in the data and demonstrated powerful performance on both the train and test datasets. The model achieved $R^{2}$ value of 0.997 and 0.925 for training and testing data, respectively. Furthermore, less scatter of the GBRT predicted values of the test dataset was accomplished compared to the GRU and GP models. The actual versus GBRT predicted compressive strength of the test data is displayed in Figure 10.

### 4.2. Comparison of Model Performance

Based on the results discussed above, all developed ML models could predict the compressive strength of RAC with reasonable accuracy. However, the GRU and GBRT models demonstrated higher generalization capacity since their prediction errors for training and testing datasets were highly analogous, in contrast to the GP model. The prediction accuracy for the training dataset in the GP model was very high, while it was quite low for the testing dataset. Thus, the GP model suffers from over-fitting and lack of generalization to new unseen data. Although DL models are recognized to be more accurate on large datasets, the finely tuned GRU model, despite the relatively small dataset, reached outstanding prediction performance with high generalization capacity.

**Figure 11:**
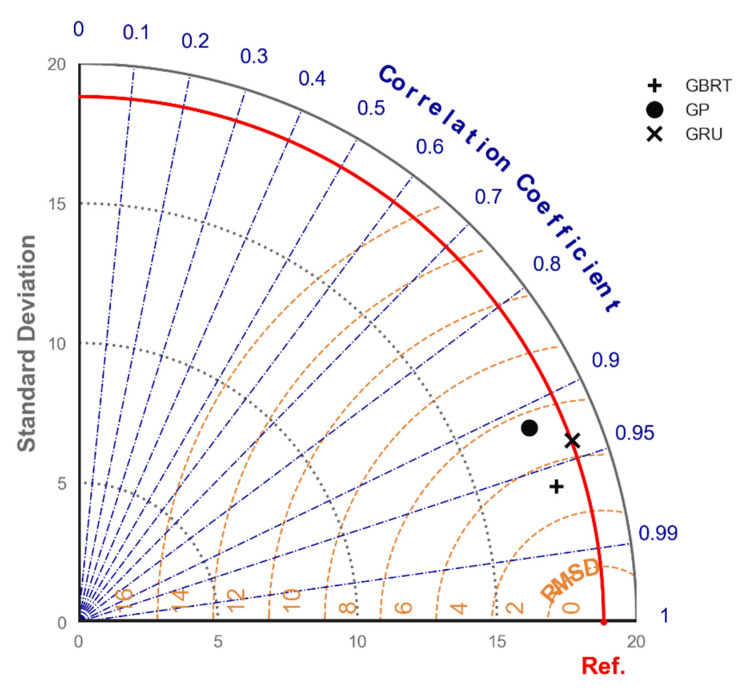
Taylor diagram comparing performance of three developed ML models.

Figure 11 illustrates the Taylor diagram of the GP, GRU and GBRT models using the RMSE, Pearson correlation and standard deviation of the predictions. The Taylor diagram suggests that the GBRT model had superior performance in terms of RMSE, whereas the GRU model provided predictions of the output with a highly correlated standard deviation to the actual observations. It is worth mentioning that the GBRT model required considerably shorter execution time for training compared to the GRU model. This comparison was performed using the same computer without mounting or connecting it to a hosted GPU. Ultimately, it was concluded that the GBRT model had the best performance and will be considered for the mixture optimization process.

### 4.3. Comparison with Previous Studies

A prime goal in ML is to create models that can accurately predict the output for new unseen data never presented to the model, i.e., achieving models that can generalize [38]. ML models generalize a phenomenon through learning the underlying principles within the training data. Hence, they are capable of generalizing when predicting sensible outputs from inputs different than those of the training dataset [27]. Testing the model on a high number of unseen data samples is the rational way to determine whether the model is generalizing or not, thus the importance of having large datasets [27]. The models proposed in the present study have demonstrated better generalization capability than those in former studies. A major reason for this superior performance is that the test dataset used in this study has more data samples than the entire datasets used in developing previous models, including Khademi et al. [15], Duan et al. [2], Deshpande et al. [11], Topçu and M. Saridemir [6], Deng et al. [16], and Naderpour et al. [1] (see Table 14). It is important to mention that the Deng et al. [16] model was not included in Table 14 because they neither reported the coefficient of determination nor the root mean squared error. However, they reported the relative error percentage, which corresponded to 6.63, 4.35, and 3.65 for the back propagation neural network, support vector machine, and convolutional neural network, respectively.

Table 14 shows the coefficient of determination and the root mean squared error of models in previous studies that predicted the compressive strength of RAC. It can be observed that models in the present study achieved better accuracy than that of Gholampour et al. [22] and Deshpande et al. [11] who used relatively large data samples. As expected, studies that reported smaller databases reached higher accuracy. For instance, Duan et al. [2] and Khademi et al. [15] used 168 and 257 samples, respectively. The reported accuracy was 0.995 for Duan et al. [2] and 0.919 for Khademi et al. [15], both studies using ANNs. This indicates that although higher number of samples might result in a better generalized model, the accuracy can decrease, and thus accuracy metrics alone may not be enough to assess predictive models. The ability to generalize predictions beyond a limited dataset is a more important feature. Also, several models which used smaller data sets than that in the present study, including Khademi et al. [15], Duan et al. [2], Deshpande et al. [11], Topçu and Saridemir [6], Deng et al. [16], and Naderpour et al. [1], have compromised generalization capability. Furthermore, in the case of Gholampour et al. [22], the authors decided to split the available data and create two different models to predict the compressive strength of those samples corresponding to cylindrical specimens and those corresponding to cube specimens. Conversely, the present study considered the specimen type as an input feature, resulting in higher accuracy. Generally, the present study along with Dantas et al. [23] used the highest number of data. However, Dantas et al. [23] reported a coefficient of determination higher for the testing set than that for the training set, 0.971 and 0.928, respectively. This is a sign that their model was not sufficiently trained, as suggested by Gulli and Pal [37].

### 4.4. RAC Mixture Proportioning and Optimization

A PSO was coupled with the GBRT model to optimize the mixture design and predict the compressive strength of RAC, such that the most economic mixture proportion is obtained for a given compressive strength class. The optimization was performed considering the unit costs of materials presented in Table 7. Not only does the optimization process reduce the higher unit cost ingredients, but it also reduces cement in the mixture, providing both economic benefit and sustainable mixture designs with less CO_2_ emission. High upper limit of RCA was considered in the optimization to ensure maximum replacement of RCA as presented in Table 8. Although using higher portions of RCA may contradict with compressive strength requirements, the optimization was carried out to maintain highest possible recycle aggregate content along with the desired compressive strength class.

Table 9 presents the optimized mixture designs of RAC for different compressive strength classes as obtained by the PSO model. The mixture proportions were then used to predict the compressive strength using the GBRT model. Silica fume was not considered in the optimization process, and thus was set to zero when predicting the compressive strength with the GBRT model. Ultimately, considerable reduction of cost in all cases, especially for the lower compressive strength range, was achieved as outlined in Table 10. For instance, there was 25% reduction in the cost of the RAC mixture without affecting its compressive strength when the target compressive strength was 35 MPa. The optimization process demonstrated the outstanding capability of the PSO-GBRT model in capturing complex relationships within the data to select the best mixture proportions, while maintaining a similar water-to-cement ratio to that of the base mixture. This can be observed for instance when considering the 25 and 30 MPa compressive strength classes in which high water-to-cement ratio was proposed with high RCA content with high water absorption capacity, as observed in experimental studies [9].

## 5. Conclusions

The present study explores deploying state-of-the-art machine learning models to predict the compressive strength of RAC and optimize its mixture design. For this purpose, one of the largest existing experimental datasets, including 1134 mixture design examples and featuring 10 attributes was built from studies in the open literature. Three advanced machine learning models, including Gaussian processes (GP), deep learning (DL), and gradient boosting regression trees (GBRT) were tuned, trained, and tested using the dataset. To guarantee that the developed models were able to generalize the compressive strength of RAC, *K*-fold cross-validation was used during the tuning process. The results show that the three models successfully captured the underlying principles contributing to the compressive strength of RAC. Furthermore, the diverse nature of the algorithms used herein proves the robustness of ML algorithms for data analysis despite the complexity within the dataset. The comparison of the models’ performance revealed that the GBRT and DL (recurrent neural network) models had superior predictive performance compared to the GP model in terms of different statistical metrics and performance indicators. Accordingly, the obtained coefficient of determination of the testing set for GBRT, DL, and GP was 0.919, 0.868, and 0.844, respectively. Furthermore, the GBRT model was coupled with PSO to create a hybrid model for optimizing the mixture design of RAC with various compressive strength classes. Accordingly, the GBRT-PSO hybrid model successfully proposed economic mixture designs that fulfill the compressive strength requirement, reduce cost, and mitigate the environmental footprint of concrete production. To further the high potential of the developed ML models, it is proposed to integrate supplementary cementitious materials, such as fly ash and blast furnace slag in the dataset, and to extend the models to also capture durability requirements of RAC in future work.

## Figures and Tables

**Table 1:** Studies on using ML techniques for prediction of RAC compressive strength.

Machine Learning Technique	No. of Samples	Ref.
Artificial neural networks, adaptive neuro-fuzzy inference system and multiple linear regression	257	[15]
Artificial neural networks	168	[2]
Artificial neural networks, model tree and non-linear regression model	257	[11]
Artificial neural networks and fuzzy logic	210	[6]
Convolutional neural networks	74	[16]
Artificial neural networks	139	[1]
Artificial neural networks	1178	[23]
Multivariate adaptive regression splines, M5 model tree and least support vector regression	650	[22]

**Table 2:** Sources of experimental data used in this study.

Authors	No. of Samples	Ref.	Authors	No. of Samples	Ref.
M. C. Limbachiya et al.	12	[46]	S. Manzi et al.	10	[47]
A. Ajdukiewicz and A. Kliszczewicz	117	[48]	A. B. Ajdukiewicz & A. T. Kliszczewicz	16	[49]
J. M. V. Gómez-Soberón	15	[50]	Y.-N. Sheen et al.	27	[51]
Y. H. Lin et al.	24	[52]	C. Thomas et al.	72	[53]
C. S. Poon et al.	36	[9]	V. A. Ulloa et al.	18	[54]
D. Matias et al.	9	[55]	W. Z. Taffese	10	[56]
M. Etxeberria et al.	4	[57]	G. Andreu and E. Miren	30	[58]
M. Etxeberria et al.	12	[59]	M. G. Beltrán et al.	9	[60]
S. C. Kou et al.	40	[61]	M. G. Beltrán et al.	8	[62]
C. S. Poon et al.	8	[63]	Ö. Çakır and Ö. Ö. Sofyanlı	27	[64]
K. Rahal	70	[65]	J. A. Carneiro et al.	2	[66]
R. Sato et al.	11	[67]	H. Dilbas et al.	12	[68]
M. Casuccio et al.	9	[69]	Z. H. Duan and C. S. Poon	26	[8]
S. C. Kou et al.	24	[70]	P. Folino and H. Xargay	4	[71]
K.-H. Yang et al.	42	[72]	F. López Gayarre et al.	14	[73]
A. Domingo-Cabo et al.	8	[74]	C. Medina et al.	16	[75]
V. Corinaldesi	10	[76]	D. Pedro et al.	18	[7]
R. Kumutha and K. Vijai	12	[77]	M. Pepe et al.	15	[78]
M. Malešev et al.	9	[79]	G. Wardeh et al.	16	[80]
G. F. Belén et al.	16	[81]	Y. Haitao and T. Shizhu	20	[82]
G. Fathifazl et al.	6	[83]	V. W. Y. Tam et al.	24	[84]
M. Chakradhara Rao et al.	16	[85]	A. S. Abdel-Hay	4	[86]
R. Somna	18	[87]	C. Zheng et al.	36	[88]
A. Abd Elhakam et al.	30	[89]	M. C. S. Nepomuceno et al.	15	[90]
A. Barbudo et al.	36	[91]	N. Mohammed et al.	12	[92]
L. Butler et al.	8	[93]	C. Thomas et al.	23	[94]
S. Ismail and M. Ramli	12	[95]	K. H. Younis and K. Pilakoutas	18	[96]
K. Kim et al.	18	[97]			

**Table 3:** Statistical characteristics of the dataset.

**Input Feature**	**Units**	**Min.**	**Max.**	**Mean**	**Standard Deviation**
Water-to-cement ratio	-	0.24	1.02	0.49	0.12
Cement content	kg/m^3^	210.00	650.00	387.60	71.36
Sand content	kg/m^3^	419.52	1010.00	691.71	131.65
Recycled aggregate content	kg/m^3^	0.00	1358.00	527.83	444.75
Gavel content	kg/m^3^	0.00	1524.00	542.94	470.19
Superplasticizer	kg/m^3^	0.00	45.00	2.63	4.53
Silica fume content	kg/m^3^	0.00	50.00	3.47	11.60
Age	Days	2.00	365.00	44.57	70.69
Specimen type	Type	1.00	5.00	2.79	1.15
**Output**	**Units**	**Min.**	**Max.**	**Mean**	**Standard Deviation**
Compressive strength	MPa	4.30	108.51	43.57	17.72

**Table 4:** Hyperparameters for Gaussian processes model.

Hyperparameter	Assigned Value
Length scale 1, $l_{1}$	0.6
Periodicity, p	16.0
Sigma naught, $\sigma_{0}$	1.9
Length scale 2, $l_{2}$	1
Nu, ν	0.5

**Table 5:** Hyperparameters for deep learning model.

Layer	Units	Activation Function	Recurrent Activation Function	Kernel Initializer	Recurrent Initializer
Gated recurrent unit	239	ReLU	Sigmoid	Random Uniform	Constant
Gated recurrent unit	238	Sigmoid	ReLU	Random Uniform	Zeros
Gated recurrent unit	217	SELU	Softsign	Constant	Zeros
Dense	1	Softplus	-	-	-

**Table 6:** Hyperparameters for the GBRT model.

Hyperparameter	Number of Estimators	Learning Rate	Min Samples Split	Min Samples Leaf	Max Depth	Max Features	Subsample
Value	315	0.44	33	17	5	7	0.98

**Table 7:** Unit price of ingredients of concrete mixtures.

Ingredient	Units	Currency	Unit Price
Water	$/kg	Canadian dollar	0.004
Cement	$/kg	Canadian dollar	0.43
Sand	$/kg	Canadian dollar	0.28
Recycled coarse aggregate	$/kg	Canadian dollar	0.20
Gavel	$/kg	Canadian dollar	0.20
Superplasticizer	$/kg	Canadian dollar	71.07
Silica fume	$/kg	Canadian dollar	2.85

**Table 8:** Bounder vectors for mixture optimization.

Input Feature	Unit	25 MPa		30 MPa		35 MPa		40 MPa	
		Upper Limit	Lower Limit	Upper Limit	Lower Limit	Upper Limit	Lower Limit	Upper Limit	Lower Limit
Water	kg/m^3^	350	200	350	190	230	160	230	160
Cement	kg/m^3^	424	290	424	292	424	323	424	280
Sand	kg/m^3^	942	650	942	650	942	720	942	750
RCA ^a^	kg/m^3^	1080	700	1080	750	1080	550	900	750
Gavel	kg/m^3^	511	50	511	50	511	100	750	220
SP ^b^	kg/m^3^	0	0	0	0	0	0	2	0.9
Age	Days	28	28	28	28	28	28	28	28
Specimen	Type	1	1	1	1	1	1	1	1

^a^ recycled coarse aggregate, ^b^ superplasticizer.

**Table 9:** Optimized mixtures.

Optimized Mix	Water	Cement	Sand	RCA ^a^	Gravel	SP ^b^	Age	ST ^c^
	(kg/m^3^)	(kg/m^3^)	(kg/m^3^)	(kg/m^3^)	(kg/m^3^)	(kg/m^3^)	Days	Type
25 MPa	246.46	296.62	701.67	711.90	155.23	0.00	28	1
30 MPa	239.56	298.52	701.67	760.33	155.23	0.00	28	1
35 MPa	181.68	327.99	759.29	566.60	193.82	0.00	28	1
40 MPa	178.83	310.45	767.23	768.92	313.78	1.23	28	1

^a^ recycled coarse aggregate, ^b^ superplasticizer, ^c^ specimen type.

**Table 10:** Comparison of optimized mixture with base mixture.

Input Feature	Units	25 MPa		30 MPa		35 MPa		40 MPa	
		Base	Opt.	Base	Opt.	Base	Opt.	Base	Opt.
Water	kg/m^3^	234.10	246.46	190.00	239.56	175.00	181.68	187.00	178.83
Cement	kg/m^3^	390.16	296.62	380.00	298.52	350.00	327.99	311.00	310.45
Sand	kg/m^3^	702.30	701.67	637.00	701.67	730.00	759.29	840.00	767.23
RCA ^a^	kg/m^3^	1053.45	711.90	1123.00	760.33	989.00	566.60	0.00	768.92
Gravel	kg/m^3^	0.00	155.23	0.00	155.23	0.00	193.82	935.00	313.78
SP ^b^	kg/m^3^	0.00	0.00	0.00	0.00	1.68	0.00	1.56	1.23
Age	Days	28	28	28	28	28	28	28	28
ST ^c^	Type	1	1	1	1	1	1	1	1
F’c	MPa	25.3	25.5	30.1	29.6	36.0	35.5	40.0	39.9
Price	CAD	577.21	499.44	568.49	510.02	673.94	507.12	668.66	654.35

^a^ recycled coarse aggregate; ^b^ superplasticizer; ^d^ specimen type.

**Table 11:** Measured performance of Gaussian process model.

Random Seed and Global Performance	Set	*RMSE ^*b*^*	*MAE ^*c*^*	*R* ^2^
RS ^a^ = 59	Test	7.468	5.157	0.827
	Train	0.556	0.111	0.999
RS ^a^ = 1718	Test	7.589	5.197	0.834
	Train	0.789	0.144	0.998
RS ^a^ = 1009	Test	6.582	4.762	0.854
	Train	0.595	0.103	0.999
RS ^a^ = 3097	Test	7.492	4.875	0.841
	Train	0.680	0.135	0.998
RS ^a^ = 7	Test	6.305	4.566	0.862
	Train	1.055	0.197	0.997
Average	Test	7.087	4.911	0.844
	Train	0.735	0.138	0.998
Standard Deviation	Test	0.597	0.267	0.014
	Train	0.200	0.037	0.001

^a^ random seed; ^b^ root mean squared error; ^c^ mean absolute error.

**Table 12:** Measured performance of deep learning model.

Random Seed and Global Performance	Set	*RMSE ^*b*^*	*MAE ^*c*^*	*R* ^2^
RS ^a^ = 59	Test	7.298	4.663	0.835
	Train	3.064	2.16	0.97
RS ^a^ = 1718	Test	6.927	4.567	0.861
	Train	3.140	2.274	0.968
RS ^a^ = 1009	Test	5.778	4.106	0.888
	Train	3.172	2.316	0.969
RS ^a^ = 3097	Test	6.589	4.312	0.877
	Train	3.144	2.251	0.967
RS ^a^ = 7	Test	5.918	4.172	0.878
	Train	3.394	2.422	0.965
Average	Test	6.502	4.364	0.868
	Train	3.183	2.285	0.968
Standard Deviation	Test	0.649	0.243	0.021
	Train	0.125	0.096	0.002

^a^ random seed; ^b^ root mean squared error; ^c^ mean absolute error.

**Table 13:** Measured performance of GBRT model.

Random Seed and Global Performance	Set	RMSE ^b^	MAE ^c^	*R* ^2^
RS ^a^ = 59	Test	5.124	3.354	0.918
	Train	1.102	0.743	0.996
RS ^a^ = 1718	Test	5.359	3.698	0.917
	Train	1.008	0.710	0.996
RS ^a^ = 1009	Test	4.640	3.196	0.927
	Train	0.965	0.683	0.997
RS ^a^ = 3097	Test	5.168	3.335	0.924
	Train	0.970	0.704	0.996
RS ^a^ = 7	Test	5.087	3.398	0.911
	Train	1.052	0.748	0.996
Mean	Test	5.076	3.396	0.919
	Train	1.019	0.718	0.996
Standard Deviation	Test	0.236	0.165	0.005
	Train	0.051	0.024	0.0003

^a^ random seed; ^b^ root mean squared error; ^c^ mean absolute error.

**Table 14:** Comparison of statistical measurements with previous studies.

Machine Learning Technique	*R* ^2^	*RMSE*	No. of Samples	Ref.
Multiple linear regression	0.609	9.975	257	[15]
Artificial neural networks	0.919	4.446		
Adaptive neuro-fuzzy inference system	0.908	5.045		
Artificial neural networks	0.995	3.6804	168	[2]
Artificial neural networks	0.903	-	257	[11]
Model tree	0.757	-		
Non-linear regression model	0.740	-		
Artificial neural networks	0.998	2.395	210	[6]
Fuzzy logic	0.996	3.866		
Artificial neural networks	0.688	-	139	[1]
Artificial neural networks	0.971	-	1178	[23]
Multivariate adaptive regression splines	-	8.750	650	[22]
M5 model tree	-	8.250		
Least support vector regression	-	7.550		
Gradient Boosting ^a^	0.919	5.076	1134	-
Deep Learning ^a^	0.868	6.502		

^a^ model of the present study.
